# Localized Liver Injury During Normothermic Ex Situ Liver Perfusion Has No Impact on Short-term Liver Transplant Outcomes

**DOI:** 10.1097/TP.0000000000004970

**Published:** 2024-02-29

**Authors:** Jack L. Martin, Freya Rhodes, Sara Upponi, Yagazie Udeaja, Lisa Swift, Corina Fear, Rachel Webster, Gwilym James Webb, Michael Allison, Anna Paterson, Rohit Gaurav, Andrew J. Butler, Christopher J. E. Watson

**Affiliations:** 1 Roy Calne Transplant Unit, Cambridge University Hospitals NHS Foundation Trust, and Cambridge NIHR Biomedical Research Centre, Biomedical Campus, Cambridge, United Kingdom.; 2 Royal Free Hospital, London, United Kingdom.; 3 Department of Radiology, Biomedical Campus, University of Cambridge, Cambridge, United Kingdom.; 4 Department of Hepatology, Cambridge NIHR Biomedical Research Centre, Biomedical Campus, University of Cambridge, Cambridge, United Kingdom.; 5 Histopathology Department, Cambridge University Hospitals NHS Foundation Trust, Cambridge, United Kingdom.

## Abstract

**Background.:**

Normothermic ex situ liver perfusion (NESLiP) has the potential to increase organ utilization. Radiological evidence of localized liver injury due to compression at the time of NESLiP, termed cradle compression, is a recognized phenomenon but is poorly characterized.

**Methods.:**

A retrospective analysis of a prospectively collected database was performed of transplanted livers that underwent NESLiP and subsequently had a computed tomography performed within the first 14 d posttransplant. The primary study outcome was 1-y graft survival.

**Results.:**

Seventy livers (63%) were included in the analysis. Radiological evidence of cradle compression was observed in 21 of 70 (30%). There was no difference in rate of cradle compression between donor after circulatory death and donated after brain death donors (*P* = 0.37) or with duration of NESLiP. Univariate analysis demonstrated younger (area under the receiver operating characteristic, 0.68; *P* = 0.008; 95% confidence interval [CI], 0.55-0.82) and heavier (area under the receiver operating characteristic, 0.80; *P* < 0.001; 95% CI, 0.69-0.91) livers to be at risk of cradle compression. Only liver weight was associated with cradle compression on multivariate analysis (odds ratio, 1.003; *P* = 0.005; 95% CI, 1.001-1.005). There was no difference in 1-y graft survival (16/17 [94.1%] versus 44/48 [91.6%]; odds ratio, 0.69; *P* = 0.75; 95% CI, 0.07-6.62).

**Conclusions.:**

This is the first study assessing the impact of cradle compression on outcome. We have identified increased donor liver weight and younger age as risk factors for the development of this phenomenon. Increasing utilization of NESLiP will result in the increased incidence of cradle compression but the apparent absence of long-term sequelae is reassuring. Routine postoperative axial imaging may be warranted.

## INTRODUCTION

Normothermic ex situ liver perfusion (NESLiP) has emerged as a promising technique for viability assessment of marginal grafts and for minimizing cold ischemic time (CIT) that might otherwise have deleterious consequences on liver function after transplantation.^1-4^ A number of liver perfusion devices enable ex situ perfusion of liver grafts with an oxygenated blood-based perfusate at normal body temperature including the Liver Assist (XVIVO, The Netherlands), the *metra* (OrganOx, Oxford, United Kingdom), and the Organ Care System Liver (Transmedics, United States).^5-9^ These perfuse the graft with the liver lying on its anterior and cranial surface in a plastic cradle. The early pioneers of liver perfusion observed that simple perfusion could result in heterogeneous perfusion.^10^ More recently, the compression of the liver parenchyma that results from placing livers in this unanatomical dependent position has been shown to produce characteristic posttransplant changes on computed tomography (CT) and these have been termed a “cradle sign.”^9-11^ To address concerns about this compressive effect on liver parenchyma, we examined the incidence of this phenomenon and its impact on immediate and longer-term graft function.

## MATERIALS AND METHODS

We performed a retrospective analysis of prospectively collected data from transplanted livers from November 1, 2018, to December 31, 2021, that underwent NESLiP at our institution. Livers donated after brain death (DBD) and donor after circulatory death (DCD) underwent NESLiP at the discretion of the implanting surgeon for viability assessment or to minimize CIT for logistical or recipient reasons. Livers were cold preserved with University of Wisconsin solution at the time of retrieval, with some DCD donor livers undergoing a period of normothermic regional perfusion (NRP) beforehand. NESLiP was performed in “back-to-base” mode using the OrganOx *metra* as described elsewhere.^3^ Immediately before NESLiP, livers were flushed with compound sodium lactate (Hartmann’s solution, Baxter, United Kingdom).

All patients received standard postoperative care. CT scans were performed in the posttransplant period when indicated by clinical events; protocol CT imaging was not undertaken. Patients who underwent a CT within the first 14 d posttransplant were included in the study. These CTs were retrospectively reviewed.

The primary outcome was 1-y graft survival. Secondary endpoints were 7-d posttransplant peak alanine aminotransferase (ALT), incidence of cholangiopathy, and graft and patient survival. The study was exempt from institutional review board approval.

### Cradle Compression

Cradle compression was defined as the presence of heterogeneous opacification on a contrast CT scan performed within 14 d of transplantation that involved but was not limited to segment VII and VIII in allografts that had been subjected to NESLiP. The radiological perfusion abnormality ranged from a linear defect to diffuse contusion.

### Cholangiopathy

Magnetic resonance cholangiopancreatography (MRCP) was undertaken to investigate unexplained cholestatic liver chemistry or cholangitis; routine MRCP was not performed. A nonanastomotic biliary stricture was defined as an irregularity or narrowing of the lumen of intrahepatic or extrahepatic donor bile duct proximal to the biliary anastomosis demonstrated by MRCP, in the presence of a patent hepatic artery.

### Statistical Analysis

Chi-square test was used for the comparison of categorical variables, and Mann-Whitney *U* test or Student *t* test for continuous data depending on data distribution. Multiple logistic regression was applied to compare the impact of donor liver variables on the presence or absence of cradle compression. The key variables selected for the regression analyses were those that were of perceived importance, with *P* < 0.25 in the univariate analyses.

Area under the receiver operating characteristic (AUROC) was used to compare performance of variables in predicting cradle compression, and Youden index was calculated from the AUROC curve to identify an optimal donor associated with cradle compression.

The impact of cradle compression on survival was analyzed using logistic regression, and Kaplan-Meier survival curves were plotted for 1-y graft and recipient survival in participants with and without cradle compression. Differences in survival curves were analyzed by the log-rank test.

All *P* were 2-sided and significance set at <0.05. Data were analyzed using SPSS software (Version 27.0; IBM Corp, Armonk, NY), apart from confidence intervals (CIs) for percentage differences, which were performed using MedCalc statistical software.

## RESULTS

Between November 1, 2018, and December 31, 2021, 112 livers were transplanted after a period of NESLiP. In the postoperative period, 70 (63%) of the recipients had a contrast CT scan of the abdomen within the first 14 d posttransplant. Of these 70 recipients, 41 (59%) received livers from DCD donors, of which 17 had been recovered using in situ NRP before NESLiP. Radiological evidence of cradle compression was an incidental finding in 21 of 70 patients (30%) (Table 1).

**TABLE 1.** Comparison of characteristics of donor livers with and without cradle compression

Donor liver and recipient characteristics	Cradle compression present n = 21 (30%)	Cradle compression absent n = 49 (70%)	*P* value
Donor age (IQR)	38 (31–50)	53 (42–61)	95% CI, 2-18 (*P* = 0.018)
Donor age minimum–maximum	20–63	17–78	
DCD (%)	14 (67)	27 (55)	NS (*P* = 0.368)
DBD (%)	7 (33)	22 (45)	NS (*P* = 0.368)
NRP (%)	5 (24)	7 (14)	NS (*P* = 0.489)
NESLiP, min (SD)	529 ± 142	518 ± 211	NS (*P* = 0.19)
CIT, min (IQR)	387 (328–440)	418 (373–508)	95% CI, 2-93 (*P* = 0.036)
Donor liver weight, g (IQR)	1842 (1555–2155)	1464 (1260–1652)^*a*^	95% CI, 226-600 (*P* < 0.001)
ALT day 7 peak (IQR)	722 (458–1655)	389 (173–672)	95% CI, 90-650 (*P* = 0.008)
1-y graft survival	(16/17)^*b*^	(44/48)^*c*^	NS
1-y recipient survival	(16/17)^*b*^	(44/48)^*c*^	NS

a n = 1 with missing data.

b n = 4 with missing data.

c n = 1 with missing data.

ALT, alanine aminotransferase; CI, confidence interval; CIT, cold ischemic time; DBD, donor after brain death; DCD, donor after circulatory death; IQR, interquartile range; NESLiP, normothermic ex situ liver perfusion; NRP, normothermic regional perfusion.

### Factors Affecting Cradle Compression

There was no significant difference in the incidence of cradle compression in livers from DCD and DBD donors (*P* = 0.37). There was significant negative correlation between donor liver weight and age with younger donors having heavier livers (ρ = –3.29; *P* = 0.006).

**FIGURE 1.**
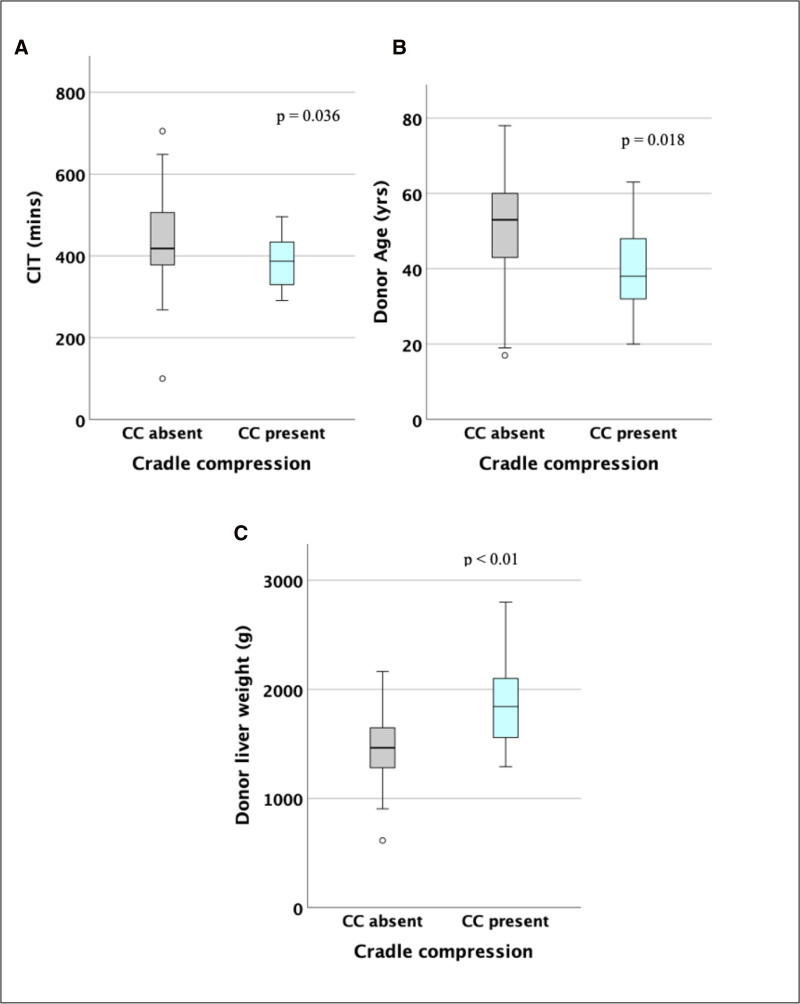
Donor characteristics associated with cradle compression. Box plots of CIT (A), donor age (B), and donor liver weight (C) against presence of CC. CC, cradle compression; CIT, cold ischemic time.

On univariate analysis, both young age (AUROC, 0.68; *P* = 0.008; 95% CI, 0.55-0.82) and high donor liver weight (AUROC, 0.80; *P* < 0.001; 95% CI, 0.69-0.91) were associated with cradle compression, whereas CIT, previous NRP, and NESLiP duration were not (Figure 1).

On multivariate analysis, when factoring in donor age, donor liver weight, duration of NESLiP, and CIT, only donor liver weight was associated with cradle compression (odds ratio [OR], 1.003; *P* = 0.005; 95% CI, 1.001-1.005) (Table 2). A Youden index-derived liver donor weight threshold of 1506 g could be used to predict cradle compression with a sensitivity of 95% (OR, 0.044; *P* = 0.003; 95% CI, 0.005-0.355) (**Table S1** and **Figure S1, SDC**, http://links.lww.com/TP/D8).

**TABLE 2.** Multiple logistic regression analysis examining impact of donor liver parameters on development of cradle compression

Variable	Significance	OR (95% CI)
CIT (min)	0.386	0.997 (0.990-1.004)
Donor liver weight (g)	0.002	1.003 (1.001-1.006)
Donor age	0.224	0.973 (0.930-1.017)
NESLiP duration (min)	0.596	0.999 (0.995-1.003)
Constant	0.190	0.030

Variable(s) entered on step 1: CIT (min), donor liver weight (g), donor age, and NESLiP duration (min).

CI, confidence interval; CIT, cold ischemic time; NESLiP, normothermic ex situ liver perfusion; OR, odds ratio.

There was a significant increase in the peak ALT (days 1–7) of recipients of livers with radiological evidence of cradle compression (722 U/L versus 389 U/L; *P* = 0.008; 95% CI, 90-650) (Table 1).

**FIGURE 2.**
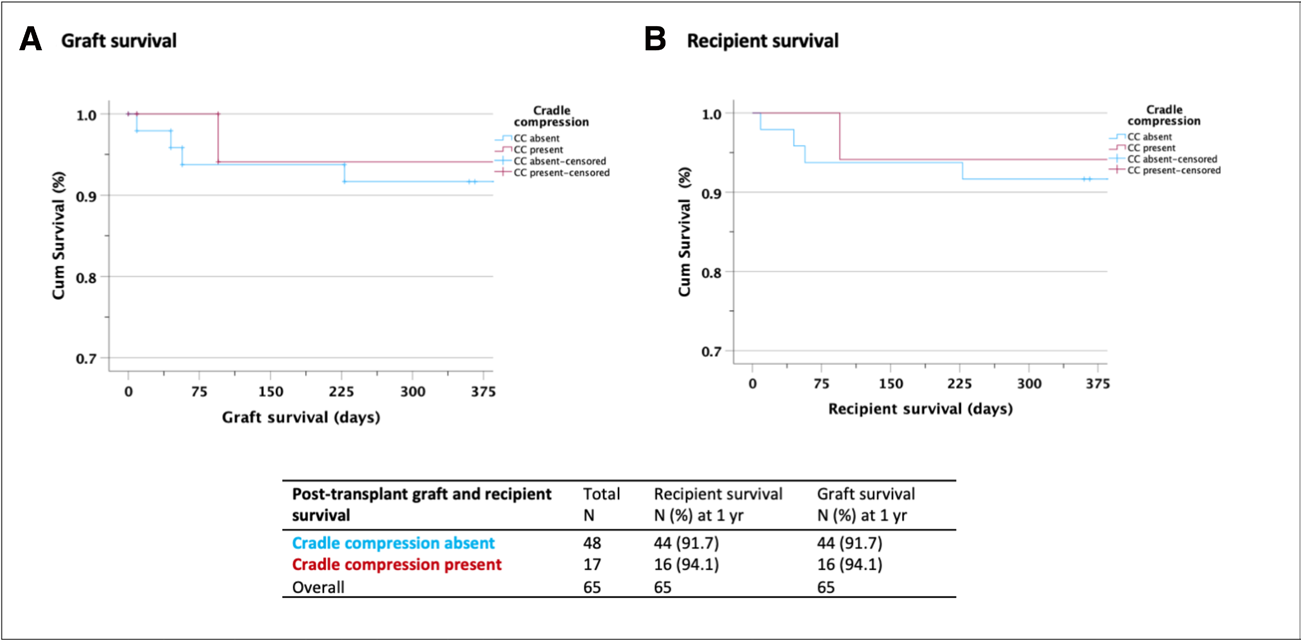
Cradle compression and 1-year graft and recipient survival. A and B, Kaplan-Meier curves of 1-y graft and recipient survival according to presence of CC. CC, cradle compression.

Data for 1-y graft and recipient survival were available for 65 of 70 recipients. Cradle compression was not associated with inferior 1-y graft survival (16/17 [94.1%] versus 44/48 [91.6%]; OR, 0.69; *P* = 0.75; 95% CI, 0.07-6.62). The results were identical for 1-y recipient survival (Figure 2A and B).

There was no difference in the incidence of cholangiopathy (3/17 [17.6%] versus 11/35 [31.4%]) without cradle compression (OR, 0.56; *P* = 0.42; 95% CI, 0.14-2.28).

## DISCUSSION

This is the first study, to our knowledge, that assesses the impact of cradle compression on outcome or to characterize risk factors for this phenomenon. We have demonstrated that cradle compression was an incidental radiological finding in 30% of liver grafts that underwent normothermic perfusion before transplantation. On univariate analysis, we identified increased donor liver weight and younger donor livers as potential risk factors in the development of cradle compression with increased donor liver weight remaining a significant risk factor with multivariate analysis. There was no difference in the incidence of cradle compression with DBD or DCD livers.

In this case-control study, cradle compression was not associated with inferior early patient or graft outcomes at 1 y, and there was no difference in the incidence of cholangiopathy between the groups.

**FIGURE 3.**
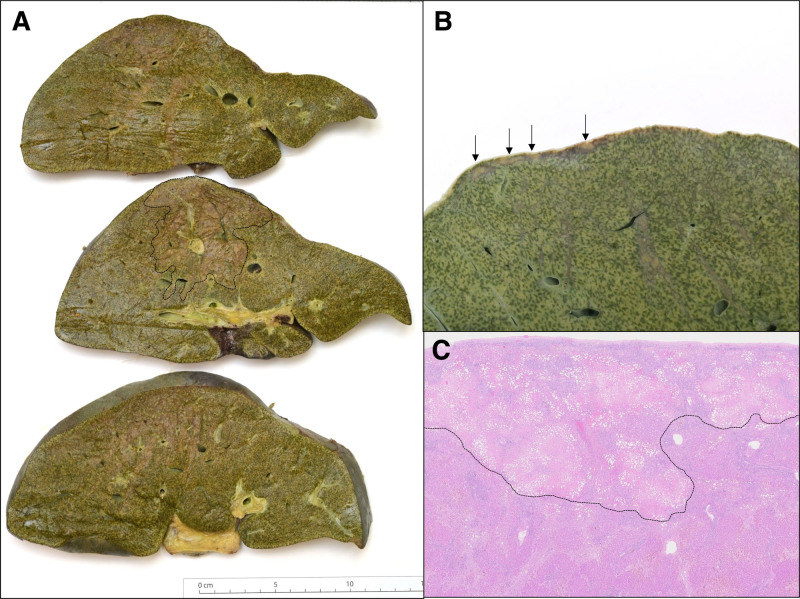
Histological specimens from a liver with evidence of cradle compression. Histological specimen from a transplanted liver that was explanted in the early posttransplant period (with super-urgent transplantation performed in the recipient) for an unrelated cause (hepatic artery thrombosis). Histology demonstrating cradle compression with radiological evidence linear defects and contusion diffusion. A, Overview of 3 sections of the liver showing cradle compression area of injury (area of cradle compression in middle section marked by dotted line). B, Image of the subcapsular injury (linear defect highlighted by arrows). C, Linear defect demonstrating microscopic features of a subcapsular band of necrosis (marked by dotted line).

We believe that the pathophysiology underlying this phenomenon is one of impaired portal venous inflow to the dependent liver while the organ is undergoing NESLiP. Moreover, the changes would appear to occur very early during NESLiP at a time when vascular resistance is high and not dependent on the duration of perfusion. While donor liver weight is a dominant factor, there are other factors that likely contribute to the phenomenon, which this analysis fails to identify. We have corroborated the radiological findings with histology in an isolated case where the recipient of a liver transplanted with radiological evidence of cradle compression required super-urgent transplantation for a cause unrelated to cradle compression (hepatic artery thrombosis). Histologically, the diffuse contusion is associated with necrosis of the involved hepatic parenchyma but with preserved portal triads. The linear defects are subcapsular bands of necrosis (Figure 3A–C).

In this study, there was a negative correlation between donor age and donor liver weight with younger donors having significantly heavier livers. This negative correlation between donor age and donor liver weight may explain the failure of the significant protective effect of increased donor age observed with univariate analysis to be maintained with multivariate analysis. Interestingly, the median age of donors from early randomized trials of NESLiP was higher (median: 56 y)^5^ than in our cohort of cradle compression cases, which may have been protective in that cohort, as might the fact the liver was placed on the *metra* at the donor hospital.^5^ Sparing of older livers could be explained by the greater propensity toward fibrosis with a stiffer liver less likely to be compressible, so preserving portal venous flow. It is interesting that we have not demonstrated localized segmental biliary complications as a late complication of this phenomenon, and this may be related to preserved arterial flow during perfusion.

There are a number of limitations to this study. It is a retrospective analysis and 30% of recipients in this cohort did not have a contrast CT performed within the first 14 d of transplant. The introduction of protocol CTs following NESLiP would be required to ascertain the true incidence of cradle compression. Moreover, while we have not seen localized biliary complications associated with this phenomenon, we did not perform regular protocol MRCPs in this cohort, and therefore, cholangiopathy may also be under-reported, albeit would be asymptomatic if present.

We do not routinely assess donor liver steatosis objectively before transplantation. While there may be a correlation between liver weight and steatosis the direct correlation between steatosis and cradle compression has not been examined by this study.

This retrospective analysis has identified a phenomenon that is more common than perhaps anecdotally appreciated. Although the radiological appearances can be alarming, reassuringly, they appear to have no long-term implications on graft function and appear to resolve over time. It is important to recognize this phenomenon as it may have implications in early postoperative care including the interpretation of transaminases. In addition, there is a possibility that these areas of parenchymal necrosis are at risk of infection. This has not been observed in our series but this may reflect posttransplant antimicrobial prophylaxis or preserved hepatic arterial flow.

NESLiP is increasingly being used for a variety of reasons, including viability assessment and logistics. The relatively high incidence we report herein could support the use of routine early postoperative cross-sectional imaging in at-risk recipients of donor livers that weigh >1.5 kg which underwent NESLiP to ensure accurate recognition and classification of cradle compression, although the apparent absence of long-term sequelae is reassuring. In assessing this phenomenon with protocol CTs, we would advocate the use of both an arterial and portal venous phase. Future design of devices should consider methods to mitigate this phenomenon, perhaps by introducing dynamic support of the liver during perfusion.

**FIGURE 4.**
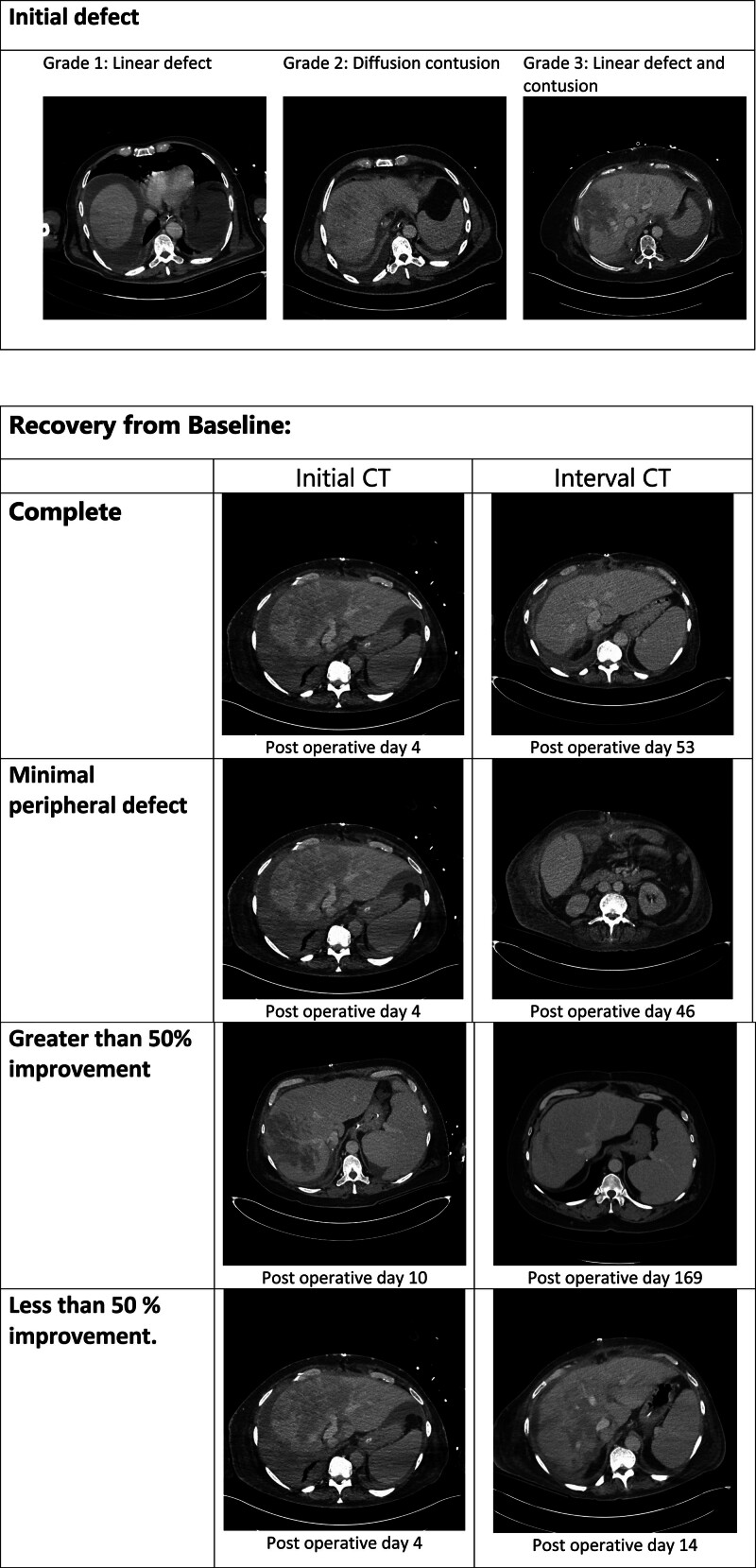
3C classification of cradle compression. Representative images demonstrating the radiological appearances used in the 3C classification of cradle compression. Patients did not undergo protocol CTs during the study period. The recovery from baseline images are from different patients. The CT scans demonstrating <50% improvement, a linear defect and complete resolution are taken from a single patient who had multiple interval postoperative scans. The >50% improvement is from a different patient. 3C, Cambridge Cradle Compression; CT, computed tomography.

To better categorize this phenomenon, we propose a scoring system, the Cambridge Cradle Compression score to classify severity and aid reporting in future studies (Figure 4). In this classification, the initial postoperative CT findings from a CT performed within 14 d of the transplant are classified as either a linear defect (grade 1), diffuse contusion (grade 2), or both a linear defect and diffuse contusion (grade 3) that involves (but is not limited to) segment VII and VIII. The recovery from baseline CT is assessed on an interval contrast CT and is classified as follows: complete recovery (R1), minimal peripheral defect (R2), >50% recovery of parenchymal opacification (R3), <50% recovery of parenchymal opacification (R4), and unchanged appearances (R5). Routine, interval follow-up CTs in patients with perfusion abnormalities were not performed. In this cohort, the initial findings had the following distribution: grade 1: 38%, grade 2: 24%, and grade 3: 38%. There was complete resolution in 69% (11/16) in those who had interval scans. The median time at which resolution was seen on subsequent CT imaging in these patients was 53 d. Of the remaining patients, 19% (3/16) did not have an interval scan and 12% (2/16) had persistent perfusion abnormalities on scans performed at 121 and 130 d posttransplant. Post hoc analysis demonstrates an association between the recipient peak ALT (days 1–7) and the severity of cradle compression. There was a significant difference in mean peak ALT between grades 1 and 3 (*P* = 0.034) (**Figure S2**, **SDC**, http://links.lww.com/TP/D8). The numbers in these groups were small and require validation with future analyses.

In summary, we have demonstrated that up to 30% of liver grafts demonstrate radiologically identifiable perfusion defects following NESLiP, but these do not impact long-term graft or patient survival. With the expected increase in the use of machine perfusion in liver transplantation, we believed that robust recognition and classification of this phenomenon is important to ensure that longer-term follow-up corroborates our findings and have therefore proposed the Cambridge Cradle Compression score.

Furthermore, future design of NESLiP devices should consider modifying the currently used technique of perfusing liver grafts in a fixed plastic cradle, lying the liver on its anterior and cranial surface. A more dynamic system that retains the ease of cannulating and controlling the inflow and outflow to the liver while continuously changing the dependent area of the liver may ameliorate this phenomenon and some authors have demonstrated success with such techniques.^12^ Further work to characterize the factors that predispose livers to cradle compression is required.

## Supplementary Material


