# Supplementary material for: Localized Liver Injury During Normothermic Ex Situ Liver Perfusion Has No Impact on Short-term Liver Transplant Outcomes
Source: Transplantation. 2024 Feb 29;108(6):1403–9. doi: 10.1097/TP.0000000000004970 (PMC11115454; doi:10.1097/TP.0000000000004970)

Supplemental Digital Content

Localised liver injury during normothermic ex situ liver perfusion has no impact on short term liver transplant outcomes

Authors

Jack L Martin, PhD[1*#]

Freya Rhodes, PhD[2#]

Sara Upponi, MPhil[3]

Yagazie Udeaja, MBBS[3]

Lisa Swift[1]

Corina Fear[1]

Rachel Webster[1]

Gwilym James Webb, PhD[4]

Michael Allison, PhD[4]

Anna Paterson, PhD[5]

Rohit Gaurav, MS[1]

Andrew J Butler, MChir [1]

Christopher JE Watson, MD[1]

Table S1: Area under the receiver operating characteristic (ROC) curve of performance of donor liver characteristics in predicting cradle compression

Area Under the ROC Curve

Test Result Variable(s)	Area	Std. Error	Sig.	95% CI Lower Bound	95% CI Upper Bound
Donor Age	0.684	0.069	0.016	.549	.819
CIT (mins)	0.650	0.068	0.049	.517	.783
NESLiP duration (mins)	0.536	0.072	0.0634	.396	.676
Donor liver weight (g)	0.797	0.056	0.000	.687	.907
Feng DRI	0.638	0.72	0.070	0.497	0.779

Figure S1: ROC curve of key variables with p values < 0.25 in the univariate analysis. Youden's index calculated from area under ROC curve identifying the donor liver weight that predicts cradle compression with a sensitivity of 95%

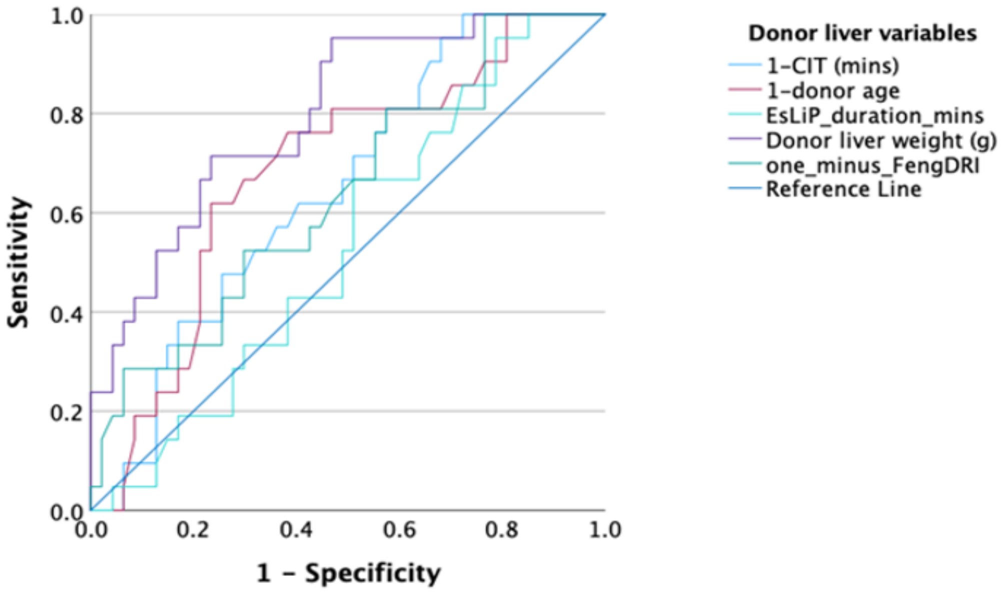

Classifier Evaluation Metrics

Test Result Variable(s)	Gini Index	K-S Statistics Max K-S[a]	Cutoff[b]
1-CIT (mins)	.301	.277	-497.50
1-donor age	.368	.385	-41.50
EsLiP_duration_mins	.072	.165	356.50
Donor liver weight (g)	.595	.484	1505.50
one_minus_FengDRI	.277	.235	-1.3100

a. The maximum Kolmogorov-Smirnov (K-S) metric. Also the maximum value of Youden's index.

Figure S2: Boxplot of peak ALT (day 1 to 7) in different 3C score grades

There was a significant difference in the median peak ALT (day 1-7) between grades 1 and 3 (475 (IQR 237-772) vs 1535 (IQR 613-3064). Grade 1: n = 8; grade 2: n = 5, grade 3: n = 7. Pairwise comparison between Grade 1 and 3 with Kruskall-Wallis ANOVA * P < 0.05.

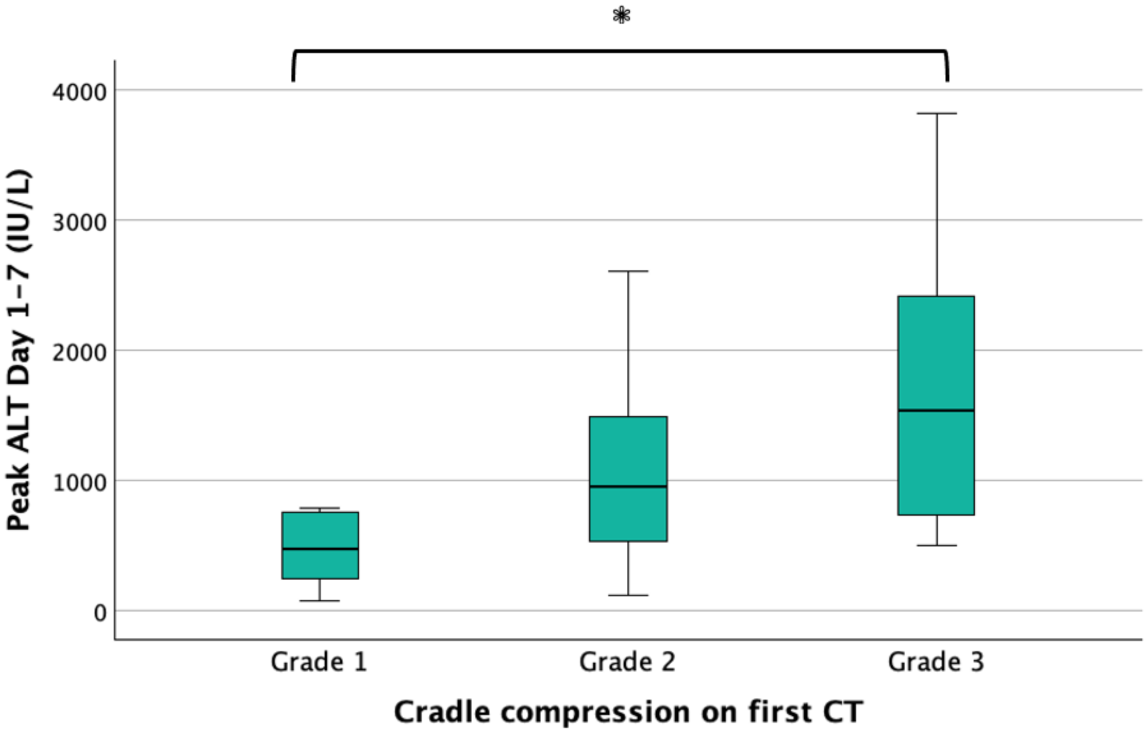

Supplement: Supplementary file 1 [file tpa-108-1403-s001.pdf]
